# Global similarity, and some key differences, in the metagenomes of Swedish varroa-surviving and varroa-susceptible honeybees

**DOI:** 10.1038/s41598-021-02652-x

**Published:** 2021-12-01

**Authors:** Srinivas Thaduri, Srisailam Marupakula, Olle Terenius, Piero Onorati, Christian Tellgren-Roth, Barbara Locke, Joachim R. de Miranda

**Affiliations:** 1 Department of Ecology, Swedish University of Agricultural Sciences, 750-07 Uppsala, Sweden; 2 Department of Forestry Mycology and Plant Pathology, Swedish University of Agricultural Sciences, 750-07 Uppsala, Sweden; 3 Department of Cellular and Molecular Biology, BioMedical Centre, Uppsala University, Husargatan 3, 751-24 Uppsala, Sweden; 4 SciLife Lab, BioMedical Centre, Uppsala University, 751 08 Uppsala, Sweden

**Keywords:** Entomology, Metagenomics, Metagenomics

## Abstract

There is increasing evidence that honeybees (*Apis mellifera* L.) can adapt naturally to survive *Varroa destructor*, the primary cause of colony mortality world-wide. Most of the adaptive traits of naturally varroa-surviving honeybees concern varroa reproduction. Here we investigate whether factors in the honeybee metagenome also contribute to this survival. The quantitative and qualitative composition of the bacterial and viral metagenome fluctuated greatly during the active season, but with little overall difference between varroa-surviving and varroa-susceptible colonies. The main exceptions were *Bartonella apis* and sacbrood virus, particularly during early spring and autumn. *Bombella apis* was also strongly associated with early and late season, though equally for all colonies. All three affect colony protein management and metabolism. Lake Sinai virus was more abundant in varroa-surviving colonies during the summer. Lake Sinai virus and deformed wing virus also showed a tendency towards seasonal genetic change, but without any distinction between varroa-surviving and varroa-susceptible colonies. Whether the changes in these taxa contribute to survival or reflect demographic differences between the colonies (or both) remains unclear.

## Introduction

The Western honeybee, *Apis mellifera*, has a worldwide distribution and plays a major role in pollination and food production^1^. Over the last few decades, large-scale managed honeybee colony losses have been reported in North America and Europe^2^, which has had a major effect on beekeeping and pollination industries^3^. There is no doubt that the ectoparasitic varroa mite (*Varroa destructor)* has been the principal driver of honeybee colony losses ever since it adapted from its original host, *Apis cerana*, to its new host *Apis mellifera* around one century ago^4^. Despite decades of research, varroa is still the primary management concern in beekeeping worldwide with the management practices still heavily focused on chemical control of the mite population^5,6^. However, it has also become increasingly evident that *Apis mellifera* as a species has the adaptive potential to reduce the impact of varroa on colony health and mortality, through either natural or artificial selection^6,7^. There are number of well-established honeybee populations in North America and Europe that have survived for more than 17 years without mite control^7,8^ and more such populations are identified continuously^9^. One of the best characterised of these populations is located on an isolated peninsula on Gotland, a Swedish island in the Baltic sea^10^, which has developed a number of adaptive traits that limit varroa reproduction^11,12^ and viral pathogen loads^13,14^, thereby reducing colony mortality. Research has shown that these adaptive traits are linked to the bee population rather than the mite population^15^; that they can be inherited^16^ and selected for^8,12^. Some of these traits have also been incorporated into honeybee breeding programmes^4,12^ in order to reduce both the impact of varroa, and the beekeeping world’s dependency on chemical treatments^6^. Many of these traits involve either complex, delicate interactions between mite and honeybee development, certain subcomponents of hygienic behaviour^11,12^, or are broad, catch-all traits such as colony survival. It is therefore not surprising that the genetic base underlying these traits is equally complex and broad, dominated by epistatic and interactive effects^12,17–19^. However, even though these traits can be inherited, it is not at all clear how much of this is through the honeybee genome, the epigenome or the metagenome, or how these different genomic systems interact to generate the adaptive phenotype. Slightly forgotten in the focus on varroa-reproductive traits is the highly significant role of pathogens, especially varroa-vectored viruses^20^, in varroa-induced colony mortality^2,4^. The logical corrolary is that viruses, and maybe other members of the honeybee microbiome, could perhaps also play an important role in natural colony-level survival of varroa infestation. This is the purpose of the current study.

The honeybee metagenome consists mostly of bacteria and viruses, when counting individual infectious units, with a minor proportion belonging to the other main microbial taxa (fungi, protists and other eukaryotic symbionts). The adult worker honeybee harbors approximately 10^9^ bacterial cells, whose general composition is consistent across the globe^21,22^. The *A. mellifera* gut bacterial community is dominated by nine species clusters from the Phyla *Firmicutes*, *Actinobacteria* and *Proteobacteria*^21,23,24^. Newly emerged *A. mellifera* honeybees have very few or no gut bacteria and acquire their gut bacteria through social interactions and contact with the hive surfaces during the first few days after emergence^25^. Genomic analysis and in vitro studies reveal that the honeybee microbiome plays an important role in digestion of macromolecules^26,27^, immune function^28^, growth and development^24,29^, defense against pathogens^30–32^ and chemical exposure^33–35^, all of which are important to individual bee health and longevity^27,29^, and thus also for colony winter survival^24^. Although the taxonomic diversity of the bee microbiome is relatively constant^21,23^, its composition is quite dynamic and responsive to both social^36^ and natural environmental influences, such as foraging and land-use^37^, seasonality^22,38^ and chemical exposure^33^. Bees are also host to a large number of viruses^39^ although in practice the honeybee virome is dominated by a just handful of these^20^. Some viruses are directly transmitted by varroa and have complex virulence relationships with bees, varroa and each other through different transmission routes^20,40–42^ while others are mostly indirectly affected by varroa, especially at colony level^43^. We recently showed that while during the summer, the titres of several common bee viruses were similar for the varroa-surviving colonies and local varroa-susceptible colonies on Gotland, these titres consistely became several orders of magnitude lower in the varroa-surviving colonies, relative to the varroa-susceptible colonies, during autumn: a critical period for the winter survival of honeybee colonies in temperate regions^13,14^. The logical questions were therefore: (1) if this was a singular occurrence or a consistent feature of the varroa-surviving population, and (2) if there were other components of the honeybee microbiome that were similarly affected by, or contributing to, the natural varroa resilience phenotype developed by this isolated bee population on Gotland. We therefore repeated the seasonal survey of varroa-surviving and varroa-susceptible colonies using an improved experimental design with greater seasonal range and closer control over the local and hive (microbial) environment, queens and sample origin. The study focuses on the bacterial and viral metagenomes, with the analysis of the other microbial taxa (fungi, protists and other eukaryotic symbionts) deferred to future studies.

## Results

We tracked the seasonal fluctuations in the quantitative and qualitative composition of the bacterial and viral metagenome in 14-day old adult bees from twelve colonies headed by 1-year old queens from the naturally varroa-surviving (MR) honeybee population on Gotland (six colonies) and from a local varroa-susceptible (MS) honeybee population (six colonies). The adult bees were emerged in cages, marked and returned to their original hives prior to recapture 2 weeks later, in order to ensure that only bees of defined age and genetics were sampled. The bacterial community structure of the adult bees was determined through mass parallel 16S rDNA amplicon sequencing while the viral composition was determined through quantitative RT-qPCR and RNA sequencing of the major bee viruses circulating in the Swedish honeybee populations^13,14^.

### Bacterial microbiome community structure

**Figure 1:**
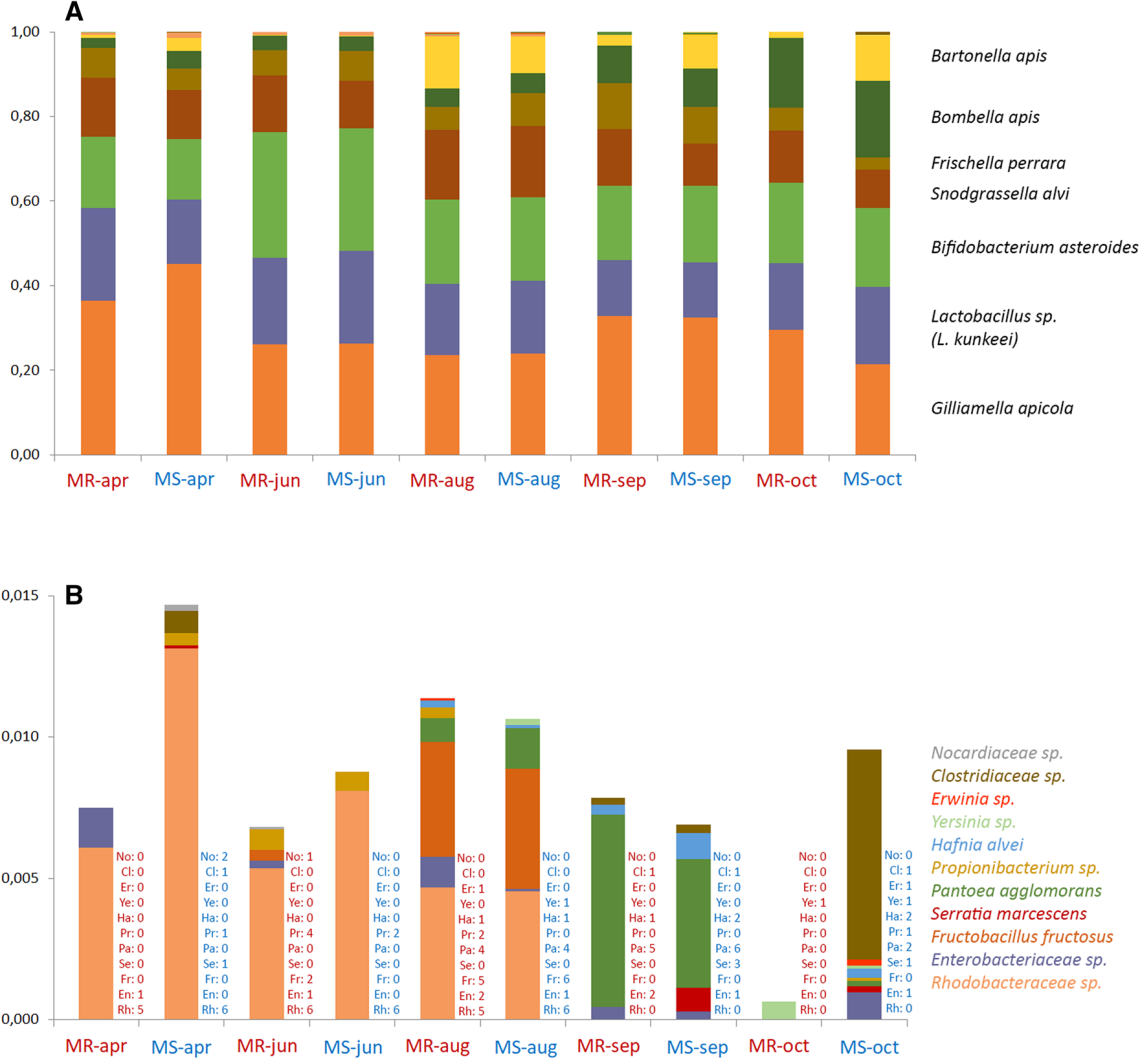
Bacterial community composition. Distribution of the most common bacterial OTUs in the MR and MS colonies during the 2015 season. Each bar represents the mean relative abundance of reads from the 16S rRNA V2 hypervariable region assigned to different bacterial OTUs, averaged over the six colonies in each of the MR (red) and MS (blue) genetic groups. (**A**) Displays the full bacterial composition, including both core and minor taxa, with the names of the most abundant taxa shown beside their stacked segment. (**B**) Highlights the relative contribution of just the 11 minor taxa, accounting for < 1.5% of the total bacterial compositiion. The numbers beside each stacked histogram indicate how many of the six colonies in the MR or MS groups contributed reads for each of the minor taxa (identified by their first two letters) while the legend shows the bacterial taxa in the order in which they are stacked in the histogram, identified by colour.

The bacterial microbiome of these samples comprised eighteen operational taxonomic units (OTU) representing eleven Classes in five bacterial Phyla (Fig. 1; Supplementary Fig. 1). More than 99% of the bacterial OTU’s belonged to seven taxa: *Gilliamella apicola*, *Snodgrassella alvi*, *Frischella perrara*, *Bombella apis* and *Bartonella apis* from the Phylum *Proteobacteria*; *Lactobacillus* spp. (mostly *L. kunkeeii*) from the Phylum *Firmicutes* and *Bifidobacteria* spp. (mostly *B. asteroides*) from Phylum *Actinobacteria* (Fig. 1A). The remaining 1% of OTU’s were distributed among eleven minor taxa (Fig. 1B) from the *Clostridia* (Phylum *Firmicutes*); the *Bacteroidia* and *Flavobacteriia* (Phylum *Bacteroidetes),* and the *Gloeobacterophycidae, Oscillatoriophycidae* and *Prochlorales* (phylum *Cyanobacteria*).

### Bacterial microbiome compositional changes

There was considerable variation in microbial composition across the five sampling occasions at all levels of classification, but with generally very similar distributions for the varroa-surviving (MR) and varroa-susceptible (MS) colonies at each sampling occasion, especially for the most abundant bacteria (*Snodgrassella*, *Gilliamella*, *Lactobacillus*, *Bifidobacterium*, *Frischella* and *Bombella*). However, some significant differences could also be identified, particularly the lower proportion of *Bartonella* in MR colonies during the early (April) and late (September, October) parts of the season (Kruskal–Wallis ANOVA:* P* = 0.014), offset by a higher proportion of *Frischella* (Kruskal–Wallis ANOVA:* P* = 0.044) and in April also of *Lactobacillus* (Kruskal–Wallis ANOVA:* P* = 0.006). Another observation is the higher abundance and diversity of minor bacterial taxa in the MS colonies during April and October (Fig. 1B). Although *Serratia* (a minor taxa and occasional bee pathogen^44^) was neither common nor abundant in these experiments, its complete absence from all MR colonies at all times was a strong driver of statistical significance between the two types of colonies, both for the individual *Serratia* comparisons and for various global microbiome biodiversity metrics.

**Figure 2:**
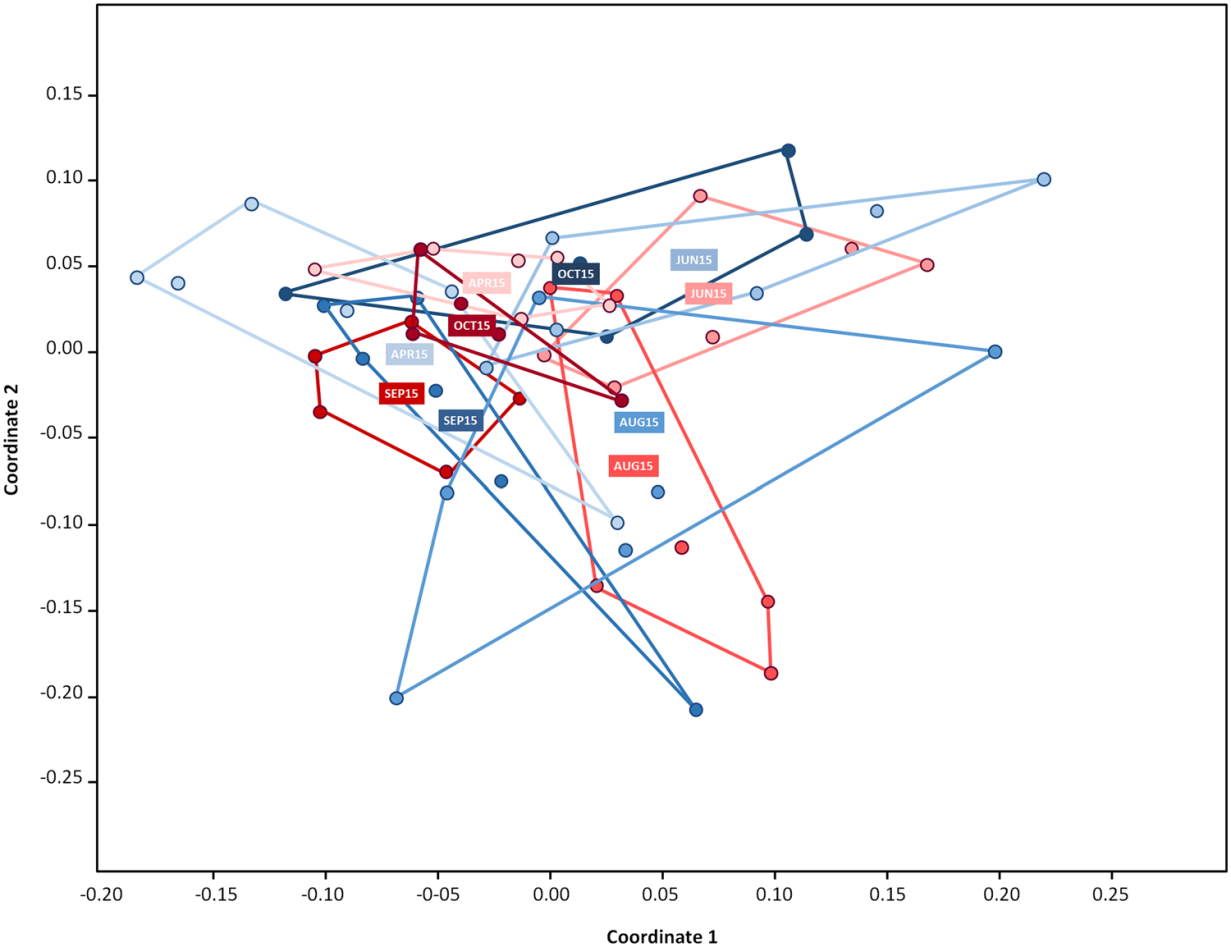
Clustering analysis bacterial composition. Two-dimensional NMDS ordinations clustering the bacterial community structures of the six MR and six MS colonies for each of the five sampling occasions. Analysis of similarity (ANOSIM) revealed statistically distinct (*P* = 0.0001) communities of bacteria for the different sampling occasions, but with overlapping structures for the MR (red) and MS (blue) colonies at each sampling occasion (increasingly darker shades of colour from April to October).

**Figure 3:**
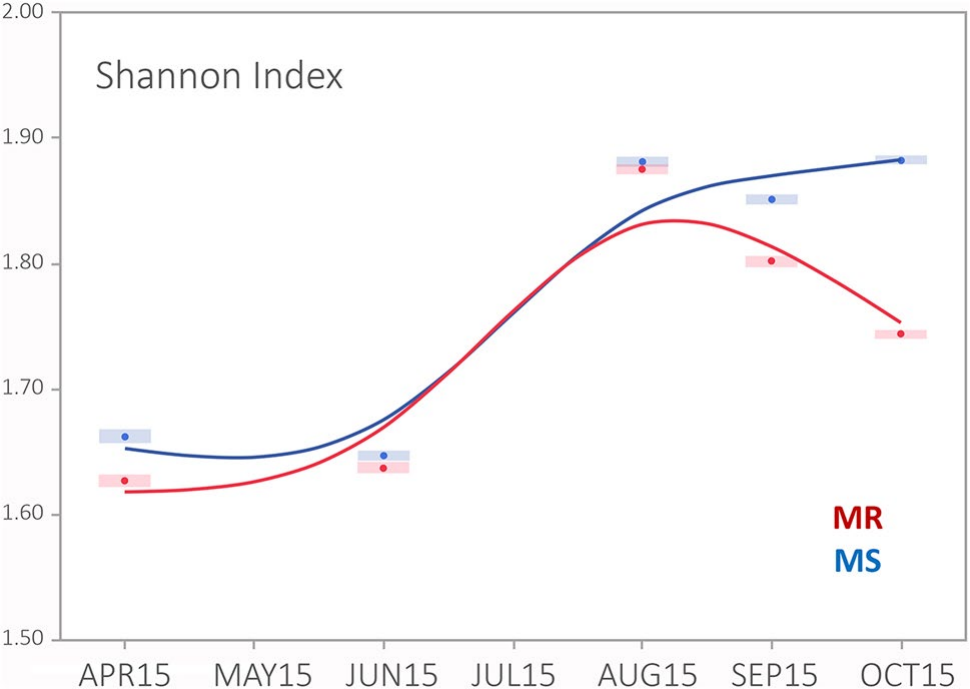
Bacterial community diversity analyses. Shannon H-index estimates for bacterial community diversity in the MR (red) and MS (blue) colonies between April and October 2015. The size of the circles represents the standard deviation for the estimate at the nodes. Similar graphs were obtained with other diversity estimates.

Biodiversity, i.e. the variety and abundance of species, has two core components: how many species are detected (richness) and how frequently they are detected (evenness)^45^. Different weighted estimates of richness and evenness are then combined into a diversity index, of which the Shannon H and Simpson 1-D are the most common^45^. There was no significant difference between the MR and MS microbiome richness estimates during the season, but there was consistent and significant difference in evenness across a range of indices, particularly during the early and late parts of the season (Supplementary Table 1). Although biodiversity measures differ in how they emphasize species richness and evenness, two of the most widely used indices, the Shannon-H and the Simpson 1-D^45^, gave very similar evaluations of the differences in bacterial biodiversity between the MR and MS colonies across the season (Supplementary Table 1). Figure 2 displays this information graphically for the Shannon-H index, which shows two trends: a general increase in microbial biodiversity as the season progresses and a divergence between the MR and MS samples in microbial diversity towards the end of the season, with a higher diversity in the MS colonies than in the MR colonies at the end of the season. This divergence is driven mostly by the higher abundance and diversity of the minor bacterial taxa (and *Bartonella*) in the MS colonies, rather than by any drastic change in the major taxa. These overall microbiome diversity measures were analysed statistically using a non-metric multidimensional scaling (NMDS) analysis with Bray–Curtis dissimilarity measure (Fig. 3). An analysis of similarity (ANOSIM) revealed that the temporal-seasonal patterns in the NMDS were significantly different for the five sampling occasions (ANOSIM: *P* = 0.0001), but not for the differences between the MR and MS colonies at each sampling occasion (Fig. 3). We followed up these scaling analyses with a post hoc non-parametric multivariate analysis of variance (NPMANOVA) involving pair-wise comparisons of MR and MS samples from the five sampling occasions across the season (Supplementary Table 2). These analyses broadly confirm that there are significant differences in microbial composition across the season^22^, for both the MR and the MS colonies, but that there is no great difference in composition between the MR and MS colonies at each sampling occasion (boxed figures), especially during the middle of the season.

### Quantitative virome dynamics

**Figure 4:**
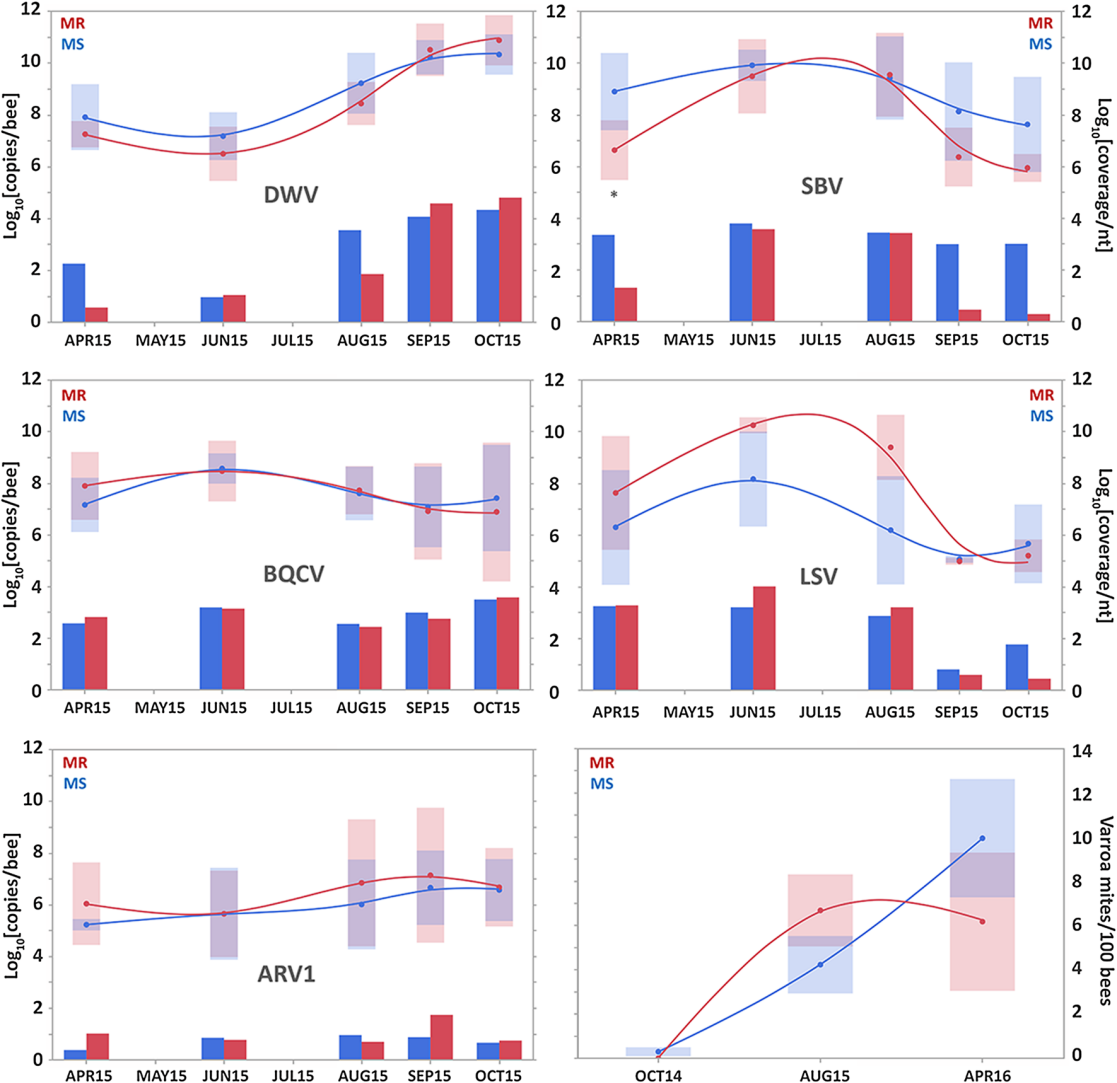
Virus titre distribution. Virus titres in adult bees from April–October 2015 season for colonies in the MR and MS honeybee colonies, as determined by RT-qPCR (line drawings, averages and confidence intervals) and RNA sequencing (histograms). Asterisks indicate that the virus titre difference between the MR and MS populations at that sampling occasion was significant at *P* < 0.05, as determined by Welch's t-test. The RT-qPCR titres are marked on the primary Y-axis (left) while the genome equivalents of the read-count data are marked in the secondary Y-axis (right). The sixth panel shows the development of the varroa phoretic infestation rates of the MR and MS colonies between October 2014 and April 2016, together with the standard deviations.

Viruses are a significant component of the bee microbial community, capable of causing serious deleterious effects on honeybee health^20,39^. There were clear trends across the season in the titres of the five major viruses found in these colonies, with different viruses peaking at different times (Fig. 4). All assays were broadly consensual for the major strains of each virus. The levels of deformed wing virus (DWV) increased by two (MS colonies) and three (MR colonies) orders of magnitude during the season, peaking in October. Apis rhabdovirus-1 (ARV-1) also increased throughout the season, by about 1 order of magnitude for both the MS and MR colonies, peaking in September. Sacbrood virus (SBV) and Lake Sinai virus (LSV) both had broad peaks during summer. SBV decreased by between one (MS colonies) and three (MR colonies) orders of magnitude by the end of autumn, while LSV decreased by between one (MS colonies) and four (MR colonies) orders of magnitude from a high in June to a low in September. Black queen cell virus (BQCV) remained relatively uniform throughout the season, with a modest peak in June. The qPCR data is largely confirmed by the normalized read-count data (Fig. 4), especially the significantly lower SBV levels in the MR colonies during early spring and autumn (Supplementary Table 3). Also of interest is the slower increase in DWV titre development for the MR colonies between April and August. The final panel shows the development of the phoretic varroa infestation rate on adult bees from October 2014 to April 2016, which is consistent with how these rates develop in similar experiments with colonies from this varroa-surviving population^8,10,14–16^. The data for the other four time-points during 2015 was unfortunately lost. The panel is therefore mostly descriptive, rather than explanatory for trends in the 2015 metagenomic data.

### Virus phylogenetic analyses

**Figure 5:**
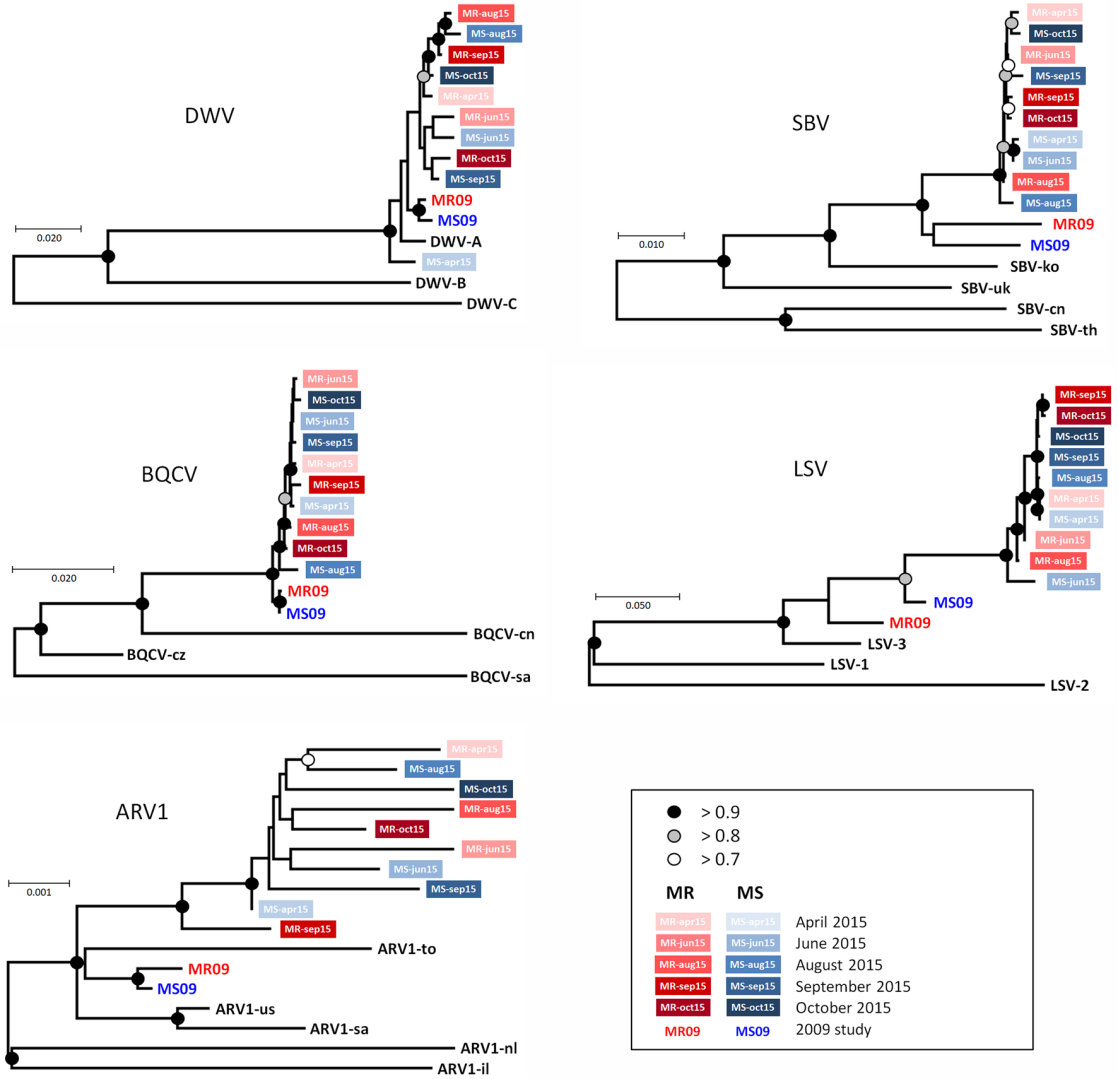
Phylogenetic analyses viral genomes. Phylogenetic analyses of the DWV, SBV, BQCV, LSV and ARV-1 consensus sequences for the MR colonies (red) and MS colonies (blue) from April to October 2015, with the seasonal sequences represented by increasingly darker shades of colour.

The full genome consensus sequences of the five viruses for the two types of colonies at each sampling occasion were determined through RNA sequencing of pooled RNA samples. These sequences were submitted to phylogenetic analyses, to determine if there were any systematic and progressive changes in the genetic composition of these viruses during the season, or in relation to the origin of the colonies. The results are shown in Fig. 5. For SBV, BQCV and ARV-1, the seasonal MR and MS samples are fairly randomly dispersed across the phylogram, without any strong pattern or affiliation and very weak support for the internal branching pattern. For DWV and LSV there appears to be a slight clustering of the late-season MR and MS sequences, which are also internally much more genetically uniform and consistent than the spring and summer samples. However, for neither DWV or LSV was there any sign of genetic differentiation between the samples from the MR colonies and the MS colonies, suggesting that the MR and MS origins or phenotypes of the colonies did not exert any strong selective pressure on the genetic composition of the main bee viruses. DWV and LSV were the most variable viruses, BQCV and ARV-1 the least variable, while SBV was moderately variable (Supplementary Fig. 2). For DWV the variability was concentrated at the 5′ end of the genome, particularly the 5′ Non-Translated Region and the Lp gene, with further moderate variability at amino acid level in capsid proteins VP2 and VP3, while for LSV the variants were concentrated in the 3′ Non-Translated Region and the intergenic regions, with further moderate variability at amino acid level in ORF-1 and the RNA dependent RNA polymerase. For both DWV and LSV the variability tends to be associated with seasonality (Fig. 5). For SBV the amino acid variability is mostly located in the 3C-protease and RNA-dependent RNA polymerase region and is less clearly associated with either seasonality or genetic origin (Fig. 5, Supplementary Fig. 2). Of the four major strains of DWV^46^, only DWV-A was recovered by the bioinformatic analyses, which is consistent with the status of DWV in Sweden at the time these experiments were conducted^13^.

## Discussion

Although *Varroa destructor* is still the primary biological threat to honeybee colony survival, especially in temperature climates, there is increasing evidence that *Apis mellifera* does posess a broad suite of biological and behavioural traits that affect the ability of the mite to reproduce^11,12^. This includes traits enabling honeybee colonies to better tolerate both the direct damage caused by varroa to pupae and adult bees^47^, as well as indirect damage caused by virus infections transmitted by the mite^4,20^. Paradoxically, some of these viruses also benefit the colony, by facilitating the detection and removal of mite-infested pupae^48^ or interfering directly with mite behaviour and reproduction^49^. This complex dynamic of beneficial and detrimental virus effects is mediated through the social functions and interactions in the colony, which means that their net impact on colony health depends on when and where they are encountered, and at what levels, in relation to the bee and mite population development^4^. Adult bees from colonies headed by queens from several European naturally varroa-surviving honeybee populations have significantly higher tolerance to experimental infection with the two main varroa-transmitted viruses, DWV and ABPV^50,51^ than those from control colonies, headed by queens from varroa-susceptible stock. Colonies from the Gotland varroa-surviving population also had much lower overall virus burden than varroa-susceptible control colonies during autumn, when winter bees are produced^13,14^. In these experiments we addressed these latter observations in greater detail, with a superior experimental design, wider seasonal range, greater control over environmental factors, better resolution of sample age and colony origin and extending the analyses to include the bacterial microbiome as well as the viral microbiome, with the analysis of the other major microbial taxa (fungi, protists and other eukaryotic symbionts) deferred to future studies.

The bacterial metagenome was investigated using the hypervariable regions of the 16S ribosomal RNA, a well-established method for screening bacterial diversity in complex samples^52^, with the V2 hypervariable region chosen as providing the most balanced distribution of the major bacterial taxa at various levels of classification^27,53^.

The honeybee bacterial microbiome is dominated by a core group of about 8–10 taxa^23,24,27^ that together comprise about 98% of the bacterial microbiome, supplemented by a large diversity of minor taxa that in this study accounted for about 1–2% of the bacterial composition throughout the bee season. *Snodgrassella alvi, Gilliamella apicola, Frischella perrara, Bartonella apis, Bombella apis* and bacteria belonging to *Lactobacillaceae* and *Bifidobacteriaceae*, were highly abundant at some point during the season, which is consistent with previous reports^21,22^. *Lactobacillaceae* and *Bifidobacteriaceae* are lactic acid fermenting bacteria that reside primarily in the honeycrop, where they contribute to the curation of nectar and the inhibition of bacterial pathogens^30^. Their relative prominence during the main nectar foraging season implies that 2-week adult bees were mostly involved in nectar foraging and processing during that time. *Bartonella apis* was the bacterial taxa that showed the greatest and most consistent differentiation between the MR and MS colonies, particularly during the early (April) and late (September, October) parts of the season, with considerably less *Bartonella* in the MR colonies than in the MS colonies at these time-points. It is also the taxa with the greatest seasonal fluctuation, disappearing almost entirely during the height of the foraging season (June) only to become relatively abundant during August and remain abundant in the MS colonies but not the MR colonies. *Bartonella apis* is part of a genus of common insect symbionts whose role is still unclear, but is possibly related to protein and nitrogen metabolism^54^. *Bombella apis* is another taxa that is strongly associated with the late season, in both the MR and MS colonies. *Bombella apis* is part of the mouth microbiome and probably involved in curing the glandular secrections fed to larvae and queens^55,56^. The relative prominence of both *Bartonella* and *Bombella* during autumn suggests that 2-week old adult bees were mostly involved in brood rearing during that time, as well as protein consumption for the MS bees. *Gilliamella apicola* and *Snodgrassella alvi* are the main bacterial species of the mid- and hindgut, whose principal roles include food metabolism, neutralization of dietary toxins and protection against gut parasites and pathogens^24,27^. *Frischella perrara* is a facultative pathogen associated with scabbing and melanization in the pylorus region connecting the mid- and hindgut^57^.

There were a number of interesting trends in the diversity and complexity of the bacterial metagenome in relation to season and colony origin. The strongest fluctuations in complexity were in relation to the seasonal sampling, with an overall increase in bacterial diversity and complexity as the season progressed. There was relatively little difference between the MR and MS colonies in these trends, except towards the end of the season, where the MS colonies had a significantly higher species richness and overall diversity than the MR colonies, and this diversity was furthermore distributed more evenly in the MR colonies. However, most of this increase in complexity concerns the presence in some of the MS colonies during certain parts of the season of several minor bacterial taxa that are absent in most of the MR colonies most of the time, but also absent some of the time in many of the MS colonies. In other words, the nature of the complexity differential is not universal either across the season or between the colony origins. Moreover, even though the rarefaction analyses quickly reached saturation, suggesting that enough reads were available for even rare taxa to be included, most of the minor taxa were detected close to the 10 read threshold for inclusion in the analyses. Therefore, even though the differences between the MR and MS colonies for some of the minor taxa and biodiversity indices are statistically significant, it is not at all clear how much of this statistical significance is biologically relevant and how much is an artefact of sampling close to the taxa inclusion threshold. With this caveat in mind, it is also important to note that an overabundance of non-core bacteria can be indicative of dysbioses associated with disease, pathogens or stress^29,31,32^. For example, *Serratia* is an opportunistic pathogen of mammals and insects, reproducing primarily in the hemolymph^44^, that has associated with varroa transmission and individual bee mortality in overwintering colonies^44^ and has recently been shown in factorial laboratory experiments to reduce individual bee life-span when interacting at very high titres with antibiotic or agrochemical exposure^34,35,44^. Another statistically significant seasonal difference between the MR and MS microbiomes concerned the genus *Frischella*, which was more abundant in the MR colonies at the end of the season. *Frischella* is a common member of the honeybee bacterial microbiome and an occasional opportunistic pathogen that can induce a scabbing phenotype in the bee gut, probably because of the induction of a melanisation pathway^28^. However, the possible biological relevance of these statistically significant differences in the minor taxa of the honeybee microbiome for varroa survival is not clear, or certain.

The second part of the study concerned the amounts and genetic composition of five common RNA viruses^13,14^. The overall results were similar to those for the bacterial microbiome, in that there were clear and parallel seasonal fluctuations in the abundances of these viruses in the MR and MS colonies. However, in contrast to our initial studies^13,14^, the difference between the MR and MS colonies in viral abundance during the season were unique and specific for each individual virus, rather than a universal reduction in the titres of all viruses towards autumn. For three of the viruses (DWV, BQCV and ARV1) there were no differences between the MR and MS colonies at any of the sampling occasions. For SBV we confirmed the 2009 observations of a steeper decline in the MR colonies during autumn and early spring, relative to the MS colonies^13,14^, while for LSV we observed both a greater reduction in titre during late autumn and an greater increase in titre during the summer months in the MR colonies, relative to the MS colonies. These unique individual profiles for the different viruses imply that the mechanisms driving the fluctuations in virus titers between MR and MS colonies are at least in part specific for each virus, rather than a universal mechanism affecting all viruses similarly. SBV is strongly associated with pollen aversion in adults, affecting both nursing^58^ and foraging^59^, resulting in accelerated age-related polyethism^58^ and, consequently, a reduced adult life-span^58,60^. A reduction in SBV therefore favours higher quality brood rearing and increased life-span of adult bees, both of which are positive for colony survival, especially since a major adaptive feature of naturally varroa-surviving honeybee populations is a reduction in brood rearing^8,10,12^, so as to limit mite reproduction^11,12^. Essentially these populations invest in fewer bees, but of higher quality, with a foraging focus on pollen rather than nectar. SBV also interferes with the replication of DWV^40^, ABPV^61^, and possibly other viruses^62^; is a significant contributing factor to DWV-related virulence and mortality^60^ in controlled infection experiments, and may furthermore also affect varroa behaviour^49^. Interference between bee viruses appears to be more evident when the viruses are injected^42,63^, compared to oral inoculation^50,51,61^. These are all significant traits for natural varroa resistance, affecting both varroa and viruses^4,20,64^. SBV is therefore also an organism that merits further investigation for its role in natural varroa survival, from both a quantitative, genetic and virulence perspective. The possible significance of the quantitative differences between MR and MS colonies in the amounts of LSV is less clear. This is mostly because we still know very little about the biology and pathology of this virus, other than that it is very common in both healthy and failing colonies, does not present obvious pathological symptoms in either individual bees or at colony level, is very variable with multiple strains divided into two main clusters (LSV-1 and LSV-2), is detected in all main honeybee compartments (head, thorax, abdomen), is particularly abundant in the intestinal tissues and can be detected in varroa mites^65^. There is circumstantial evidence that LSV-1 and LSV-2 may be molecular re-classifications of bee virus Y (BVY) and bee virus X (BVX) respectively, which were characterized using serological methods during the 1980s^66^. These viruses have similar size, shape and virus particle composition^65,67,68^, seasonal incidence^65,66^, predominantly adult-based infection^65,66^, absence of overt symptoms^65,66^ and apparent lack of any association with varroa infestation^42,65,69–75^. BVX and BVY have been positively associated in co-infection studies with, respectively, *Malphigamoeba mellifica* and *Nosema apis*: two common protozoan pathogens that significantly reduce adult bee lifespan^66^. However, neither the viruses nor the protozoans are functionally dependent on each other for infection and the association may be simply a facultative consequence of infecting the same intestinal tissues and cells. The LSV in these colonies is most similar to LSV-3, a subclade of LSV-1^65,75^ (BVY) whose incidence peaks during the summer. The most significant aspect of the LSV quantitative dynamics may well be more the enormous universal reduction in LSV titre towards autumn in both MR and MS colonies, rather than either the large relative LSV excess in MR colonies during summer, when the turnover of adult bees is naturally very high, or the small relative LSV deficit in MR colonies during autumn, when LSV titres are naturally low, and the apparent absence of LSV-2 from these colonies, whose seasonal dynamics peak during winter: a much more critical period for honeybee colony survival. We have also shown that while adult bees from naturally varroa-resilient honeybee populations (including the MR population on Gotland) were equally susceptible as non-resilient bees to laboratory oral infection with DWV and ABPV, they were far less likely to die from these infections^50,51^ Such elevated tolerance of individual bees to virus infections is clearly also an adaptive advantage for survival at colony level.

Two features common to the bacterial and viral taxa that show the greatest differentiation between the MR and MS colonies in these experiments are that they are all associated to some extend with the collecting, managing or metabolism of protein, and that the differences are particularly pronounced during the early spring and autumn, which is the most critical part of the bee season for colony survival. It should be noted that these data are specific to 2-week old adults only, and there are also large and consistent differences between the microbiomes of different-aged adults in a colony^21,22,36^. This means that the microbiological differences between the MR and MS colonies are partly subject to colony size, organization and division of labour among its adult population. The MR colonies were consistently smaller than the MS colonies throughout the experiment, which may therefore have affected some of the microbiological differences observed here. The MR colonies also reared less brood, particularly drone brood, had higher incidence of chalkbrood and were more inclined to supercede at all times during the season. These are all traits associated with small colonies that, coincidentally or not, also slow varroa population development, and are known features of these MR colonies^8,10,12,15^. In other words, many of the beneficial varroa-surviving features of the MR colonies, including possible metagenomic ones, may simply be an indirect consequence of their smaller size, similar to varroa-surviving Africanized bees^4,8,11,12^.

The metagenomic changes observed here, and presumably the functions they represent^24,26^, are also very rapid and dynamic, occurring within the 18 months of the experiment. This time-frame is also much closer to the 3–4 years it took for the original population to become fully varroa-resilient^10^ than the time-frame that would be required for genomic adaptation, especially since the selective force (survival) operates at colony (i.e. queen) level, which has an average generation time of 1–2 years^76^. The honeybee metagenome is simply a much more dynamic substrate for rapid selection than the honeybee genome, similar to transcriptomic and epigenomic changes. In other words, it is well possible that a large part of the natural adaptation of the Gotland varoa-surviving population was, and maybe still is, achieved through these more dynamic short-intermediate time-scale genetic adaptive systems, rather than through more permanent, long-term genomic adaptations. Since the environmental conditions at the start of the experiment were identical for the MR and MS colonies, with the only difference the genetic backgrounds of the queens, at least some of the metagenomic changes observed must have been through interaction with genomic or epigenomic features specific to the MR population, and that can be transferred with just the queen. What these (epi)genomic features are, where they are located on the genome and how they affect colony-level phenotypes, either directly or through the metagenome, remains to be determined.

## Material and methods

### Experimental design

The current study was designed as a more carefully controlled repeat of our previous study^14^, both to confirm (or not) the original findings and to establish what other microbial factors, if any, contributed to the enhanced survival of the varroa-surviving honeybee population on Gotland. The main improvements over the first study were that both the varroa-surviving (MR) and varroa-susceptible (MS) colonies were located in the same apiary, and were thus exposed to the same environmental conditions; that the two sets of colonies were established through splitting and re-queening host colonies, with one split receiving an MR queen and the other an MS queen, thus ensuring that the paired colonies had the same initial microbial and within-hive background; that the sampling schedule included an early spring sample, which is also a critical period for colony survival, and perhaps most importantly that the sampling method involved the release and re-capture of marked, newly emerged bees, thus controlling both the age of the sampled bees and the colony origin, with only bees found in their colony of origin 2 weeks after release included in the sample. This avoids the problem of bees drifting between colonies, which is especially problematic during autumn^77^, and the possible effect of age and age-related division of labour on the adult bee microbiome^36^, while 2 weeks of adult life ensures adequate exposure to both the within-hive and external environment. These measures ensured a broader seasonal scale and a much tighter resolution of the composition of the bee samples, and thus also of any differences between the colonies during analysis.

### Colony establishment and origin

The varroa-surviving honeybee population investigated in these studies originated in 1999 on the island of Gotland, Sweden, as a part of selection experiment on honeybee survival with *Varroa destructor* mites^8,10^. The original population was constituted to have as broad a genetic basis as possible, which was achieved by collecting 120 colonies from throughout mainland Sweden with a wide diversity of locally adapted, pure-bred and mixed-race genetic backgrounds^10^. Admixture analysis of whole genome sequence data showed the genetic composition of the population to be predominantly C lineage (*A. m. carnica/ligustica*) with significant amounts of M (*A. m. mellifera*) and O (*A. m. anatoliaca*) lineage in 2000, before adaptation, and mostly C lineage with minor levels of M lineage in 2011–2012, after adaptation (EU domestic;^78^). The MS queens were obtained from a local queen producer (Grandérs Bigårdar HB, Uppsala) and were derived from a mixed-race varroa-susceptible honeybee population, thus approximating the mixed genetic origins of the MR population^10,78^. All the new MR and MS queens were produced and mated in 2014. Eight MR colonies and eight MS colonies were established in Uppsala, Sweden on 16–17 July, 2014, in 6-frame styrofoam hives with 5 cm entrances. Each colony was started with two frames foundation, one frame brood, three frames pollen/honey, 2.5 kg Apifonda (Südzucker, Mannheim, Germany) and about 1.5 kg bees pooled from eight local colonies located > 10 km outside flight range. Each colony was supplied with either an MR or MS queen in a self-release queen cage. Queen acceptance was monitored over the next 2 weeks, with replacement queens added in case of non-acceptance, until all colonies had a laying queen of the correct provenance by 29 July, 2014. The colonies were kept in two separate groups (MR and MS) in a dedicated east-facing linear apiary 5 m from the edge of a wood (N 59.816465, E 17.652309) and 600 m from any other honeybee colonies, with 3 m between individual colonies in each group and 20 m between the group of MR colonies and the group of MS colonies. On 4 September 2014, the colonies were transferred to full-size 10 frame hives, still with reduced entrances, and were each fed a total of 24 kg of a 75% solution equimolar sucrose:fructose:glucose (Bifor®, Nordic Sugar AS, Copenhagen, Denmark) and treated with tau-fluvalinate (Apistan®; Vita-Europe, Basingstoke, England) until 2 October 2014, with no further mite control strategies applied for the remainder of the experiment. All colonies were of similar strength prior to inwintering. Of the eight MR and eight MS colonies established in 2014, six MR and six MS colonies survived the winter 2014–2015 and were included in the seasonal survey conducted during 2015. These survival rates are normal in Sweden for artificial swarms with new queens introduced during the middle of the summer^38^.

### Monitoring, bee marking and sample collection

The colonies were monitored and sampled at bi-monthly intervals between April and October 2015. Although no official colony strength assessments were made, the colonies were managed with standard beekeeping practice according to the needs of the individual colony, particularly with respect to space, to expand/contract as appropriate, and swarm prevention. Entrances were kept small throughout the year to minimize drift and robbing. The MR colonies were consistently smaller than the MS colonies throughout the year, and had a distinctly higher incidence of chalkbrood. On each sampling occasion, one frame of emerging brood was taken from each colony during the first week of the month, and incubated in a closed cage in an incubator until approximately 500 new adults had emerged. These newly emerged adults were marked individually on the thorax with a unique coloured paint mark for each colony, in order to trace individual bees to their colony of origin. The 500 marked bees were returned to their colony of origin and after 2 weeks, 30 marked bees were collected from each colony for metagenomic analysis, as well as 150–300 adult bees for determining the phoretic varroa infestation rate, using the soapy water method^79^. Unfortunately, the infestation rate data for four of the five 2015 sampling time-points was lost, leaving only data from before the experiment (October 2014), after the experiment (April 2016) and just a single time-point during the experiment (August 2015), which is insufficient for reliable assessment of the phoretic varroa infestation rate as a possible explanatory variable for trends in the 2015 metagenomic data. A composite sample of 30 bees is sufficient to detect with 95% confidence a microorganism with a 10% infection rate^80^, which applies to all five viruses studied here^14^ and the core members of the bacterial microbiome^26,27^. A total of 58 bee samples were thus collected: 30 samples from the six MS colonies sampled on five occasions and 28 samples from the MR colonies, since one MR colony died in September 2015, thereby losing two samples. After the final sampling occasion, the colonies were prepared for overwintering. By April 2016, three more MR colonies had died: one queen-less and two with unmated virgin queens. The two remaining MR colonies and all MS colonies were alive and queen-right in April 2016. The bee samples were stored at − 20 °C until nucleic acids were extracted.

### Nucleic acid extraction

Each sample of 30 adult bees was placed with 5 ml nuclease-free water in a plastic mesh bag, submerged in liquid nitrogen and homogenized with a mortar and pestle. Total DNA was extracted by a Qiacube automated extraction robot (Qiagen, Hilden, Germany) from 100 µl of each homogenate, using the DNeasy Blood & Tissue (Qiagen, Hilden, Germany) kit. DNA purity and quantity was determined using a ND 8000 UV/Vis Nano-Drop Spectrophotometer (Thermo Fisher Scientific, Waltham, MA, USA). The extracted DNA was stored at − 80 °C until further analysis. Total RNA was extracted from 100 µl of the homogenate by a QiaCube robot following the RNAeasy protocol for plants (Qiagen, Hilden, Germany). The RNA was eluted in 50 µl RNase-free water, and the RNA concentration was estimated by NanoDrop. The purified RNA was stored at − 80 °C until further processing.

### RT-qPCR virus analysis

The amounts of DWV, SBV, BQCV, LSV, ARV-1 and a honeybee internal reference gene (RP49 mRNA^63^) used for normalizing between-sample differences in RNA quantity and quality^63^, were determined by reverse transcription quantitative PCR (RT-qPCR), using the iScript One Step RT-qPCR kit (Bio-Rad, Hercules, CA, USA) with SYBR Green as the detection chemistry and the Bio-Rad CFX connect thermocycler. The reactions were conducted in 20 μl volumes containing 0.2 μM each of the forward and the reverse primers for each assay (Supplementary Table 4), 3 μl RNA, 10 μl SYBR Green RTmix and 0.4 μl iScript reverse transcriptase, with the following cycling profile: 10 min at 50 °C for cDNA synthesis, 5 min at 95 °C for inactivation of the reverse transcriptase, followed by 40 cycles of 10 s. at 95 °C for denaturation and 30 s. at 58 °C for annealing, extension and data collection. Amplification was followed immediately by a Melting Curve analysis to confirm the identity of the amplification products, by incubating at 60 s: 95 °C, 60 s 65 °C with fluorescence reading at 0.5 °C increments between 65 and 95 °C. Included in each RT-qPCR run was a ten-fold dilution series of known amounts of each target, for absolute quantification. All assays were run in duplicate, with the average Cq value retained for analysis. Instances of non-amplification (zero values) were dealt with in the data-set by substituting a value equivalent to Cq = 41 (i.e. below the limit of detection) to allow instances of non-detection to be included in the quantitative analyses post-logging^63^. The qPCR data were first screened for the presence of secondary RT-PCR products through visual inspection of the Melting Curve (MC) analyses. After the visual inspection, the average of the duplicate Cq values was converted to Starting Quantity (SQ) values through the use of the external calibration curves established by the ten-fold dilution series for each target. These data were then multiplied by the various dilution factors throughout the methodology to estimate the raw copy number of each target per bee. These raw copy numbers for each sample were subsequently normalized using the corresponding RP49 values for the samples, to correct for sample-specific differences in the quality and quantity of RNA^13,14,63^. Although the mean RP49 mRNA levels in these colonies increased slightly between spring and summer before decreasing again towards autumn, reflecting the metabolic activity of the bees during the season, there was no statistically significant difference between the MR and MS colonies in RP49 mRNA levels at any one time-point (Supplementary Fig. 3). Since the RP49 mRNA levels varied on linear scale, while the virus levels varied at logarithmic scale, the seasonal fluctuations in RP49 mRNA levels had negligible effect on data normalization using the RP49 mRNA levels as internal reference standard^63^.

### 16S rDNA gene sequencing

The preparation of bacterial 16S rDNA metagenome sequencing libraries and the Ion Torrent sequencing was performed as a service by the National Genomics Centre, SciLifeLab, Uppsala, Sweden, using their internal workflow. Briefly, two regions of the bacterial 16S rDNA containing seven hypervariable regions were amplified from each of the 58 DNA samples using the Ion16S™ Metagenomics kit (Thermo Fisher Scientific, Waltham, MA, USA). This kit contains two different sets of multiplex primers targeted to amplify different hypervariable regions of the 16S rDNA: one set for amplifying the V2, V4, and V8 regions and the other for amplifying the V3, V6, V7 and V9 regions (Supplementary Fig. 4)^52^. For each sample, equimolar amounts of the various PCR products from the two reactions were pooled and used in library construction. Before library preparation, the quality of the purified PCR products was analyzed with an Agilent 2100 Bio-analyzer (Agilent, Santa Clara, CA, USA). The sequencing libraries were prepared using Ion-Plus Fragment Library Kit (Thermo Fisher Scientific, Waltham, MA, USA) and Ion Xpress™ Barcode Adapters 1–16 Kit (Thermo Fisher Scientific, Waltham, MA, USA) for ligating the unique barcode adaptor sequences, nick-repair, and purification. The libraries were quantified with Ion Universal Library Quantitation Kit (Thermo Fisher Scientific, Waltham, MA, USA) and combined in equimolar amounts in two pools, containing the 28 MR and 30 MS samples respectively. Sequencing template was prepared from these pooled samples by emulsion PCR in Ion Sphere Particles (ISPs) using the Ion 530 kit-OT2kit and was amplified on the Ion One Touch™ 2 system thermocycler (Thermo Fisher Scientific, Waltham, MA, USA). Finally, the sequencing was performed on an Ion S5 System (Thermo Fisher Scientific, Waltham, MA, USA) on two S5 530 chips: one for the 28 samples from the MR colonies and one for the 30 samples from the MS colonies. After quality filtering, a total of 10,674,279 pair-ended reads (mean read length 235 ± 5 bp) were recovered from the 58 samples, with an average of 1,779,047 reads per variable region. The sequencing data, BAM files, were analyzed on the Ion Reporter software (Thermo Fisher Scientific, Waltham, MA, USA) using a custom designed metagenomics workflow version 5.2. The Ion reporter software consists of a bundle of bioinformatics tools that helps in the analysis of Ion PGM sequencing data. The 16S metagenomic analysis workflow, which is part of Ion Reporter software, is based on the quantitative insights into microbial ecology (QIIME) pipeline^81^. The reads were aligned against two comprehensive 16S databases: the MicroSEQ 16S rRNA reference database^82^ and the curated Greengenes database^83^. The reads were aligned against the databases using Megablast with E value 0.01. A read was assigned to a genus when the identity score of the sequence alignment was > 97%, while for species assignment, the threshold was set at > 99%.

### Selection of 16S rDNA hypervariable region

Prior to in-depth microbiome analyses, an evaluation was made of the seven 16S rDNA hypervariable regions (Supplementary Fig. 5) for their relative capacity to identify and characterize the full microbial diversity of the honeybee microbiome^53^. The characterization of the microbiota of adult worker honeybees in the six MR and six MS colonies sampled on five occasions during 2015 was carried out on reads obtained via the amplicon sequencing of seven hypervariable regions of the 16S-rRNA gene: V2, V3, V4, V6, V7, V8 and V9. However, despite the care taken to mix equimolar amounts of the amplicons, the reads were not evenly distributed among the seven hypervariable regions, with particularly the four regions of the V3–V6/7–V9 amplicon displaying highly skewed distributions (Supplementary Fig. 5A). A second analysis calculated the proportion of reads from each hypervariable region that mapped to different major bacterial families (Supplementary Fig. 5B). There was a clear difference between the variable regions in their ability to identify bacteria from different families, with V9 particularly poor in identifying bacteria other than those from the Orbaceae or Enterobacteriaceae. The V3 and V6 + V7 regions were the regions with highest total number of mapped reads, but more than 70% of these mapped to the family Lactobacillaceae. The family Lactobacillaceae also dominated in the V4 region, accounting for 61% of the mapped reads. The V2 hypervariable region displayed the greatest capacity to identify the most diverse microbiota with the least amount of skew in the read distribution (Supplementary Fig. 5B). Since the seven hypervariable 16S-rDNA regions clearly differed in their capacity to detect the complete range of bacterial families, we decided to use only the data from the V2 hypervariable region to conduct our remaining microbiota analysis, so as to minimize systemic biases due to the different resolving powers of the hypervariable regions^53^. Moreover, the V2 region has been frequently used to study the honeybee microbiome^27^, facilitating future comparative analyses.

### Bacterial metagenome bioinformatic analyses

After quality control and filtering, a total of 1,201,813 sequencing reads for the V2 hypervariable region were retained, ranging from 87,223 to 141,985 per sample. A minimum of 10 reads were required for an OTU to be included in the statistical and biodiversity analyses. The excess reads were then rarified to a constant 87,223 reads per sample, which clustered into 18 major OTUs across all samples. The rarefaction analysis also showed that the overall sampling depth of the reads across all OTU’s was sufficient, since saturation was reached for all samples (Supplementary Fig. 6). The bacterial community structure was analysed step-wise according to taxonomic scale, starting at Phylum level and progressing through Class level to Genus level of the major bacterial OTU’s.

### RNA sequencing

The RNA samples were subjected to target-free sequencing in order to analyze the changes in the genetic composition of the RNA viral metagenome between the MR and MS colonies across the season. The procedures used for preparation and sequencing the samples was similar to those used previously^14^. Total RNA was pooled in equimolar amounts across the six MR colonies and six MS colonies at each sampling occasion, resulting in five MR and five MS population-level RNA samples: one each for April, June, August, September and October 2015. The pooled RNA samples were prepared for Ion Proton RNA sequencing by the National Genomics Centre, SciLifeLab, Uppsala, Sweden, using their internal workflow. For each sample, 1 μg total RNA was first depleted from ribosomal RNA using the RiboZero rRNA depletion kit (Illumina, San Diego, CA, USA). The quality of the depleted RNA was checked using the Bioanalyzer RNA Pico chip (Agilent Technologies, Santa Clara, CA, USA), after which the RNA was fragmented with Ribonuclease III and ligated to adaptor sequences. The RNA sequencing libraries were constructed using the Ion Total RNA-Seq v2 kit and were sequenced on an Ion Proton System (Thermo Fisher Scientific, Waltham, MA, USA) using two S5 550 sequencing chips: one for the five samples from the MR colonies and one for the five samples from the MS colonies.

### Virus genome reconstruction

The sequencing produced between 27 and 32 million reads per sample (Supplementary Table 5) with an average read length of around 206 nucleotides. Reads with > 50 consecutive nucleotides mapping with > 95% identity to the major reference genomes for each of the five major viruses studied here (Supplementary Table 6) were used to reconstruct local virus reference genomes. The sequence reads were then filtered by bar-code, to allocate them to different subfractions (bins) according to sample origin. For each subfraction, the sample-specific genomes of the five viruses were reconstructed by mapping the reads to the respective local reference sequences using Tmap included in TorrentSuite 5.10.1 with ThermoFisher recommended parameters. Variants were called using the variantcaller plug-in in TorrentSuite 5.10.1. The mapped nucleotides for each virus were subsequently collapsed into consensus sequences using bcftools, with a > 5× coverage threshold for inclusion of the character, so as to neutralize any bias from sequencing errors in the phylogenetic analyses. The total number of nucleotides mapping to each virus were then normalized for the genome size of each virus and the average number of reads in each sample (Supplementary Table 5), to obtain a normalized genome equivalent for each virus in each RNA sample^84^. These figures were then multiplied by the various dilution factors incurred during RNA extraction to arrive at an estimated number of genome equivalents per bee for each of the five viruses, in each of the samples (Fig. 4).

### Virus phylogenetic analyses

The ten sample-specific consensus genome sequences for each virus were aligned to the consensus genome sequences recovered from the varroa-surviving (MR) and varroa-sensitive (MS) populations during the 2009 survey, and several major outgroup sequences^14^, using the CLUSTAL-Omega multiple alignment programme^85^ with default parameters, on the European Bioinformatics Institute website, www.ebi.ac.uk^86^. The final alignments were checked for consistency and accuracy prior to inclusion in phylogenetic analyses. The evolutionary history for each virus was inferred using the Maximum Likelihood method based on the Tamura-Nei model^87^ as implemented by MEGA-X^88^, with the trees with the highest log likelihood retained. The initial trees for the heuristic searches were obtained automatically by applying Neighbor-Joining and BioNJ algorithms to a matrix of pairwise distances estimated using the Maximum Composite Likelihood (MCL) approach, and then selecting the topology with superior log likelihood value. All positions containing gaps and missing data were excluded from the analyses. The percentage of trees in which the associated taxa clustered together was determined by bootstrap analyses involving 500 replicates. The numerical summary of the phylogenetic analyses and the accession numbers of the virus sequences involved are shown in Supplementary Table 6.

### Statistical analyses

Rarefaction curves demonstrating sample saturation (Supplementary Fig. 6) were generated using Analytic Rarefaction v1.3^89^. The Alpha diversity indices Shannon (H), Simpson (1-D), Evenness (e^H/S) and Richness (S), as well as several other biodiversity measures (Supplementary Table 1) were calculated on rarified Operational Taxonomic Unit (OTU) tables by using the RDP command line tools/PAST (Paleontological Statistics) v.3.16 software^90^. Bacterial community composition was summarized at both Phylum and Genus taxonomic levels, and the results were presented as stacked histograms generated in Microsoft Excel. Significant differences in bacterial genera between MR and MS colonies at different time points were determined by univariate, nonparametric Kruskal–Wallis tests (non-parametric ANOVA). Beta diversity community patterns were analyzed by Non-metric Multi-Dimensional Scaling (NMDS) ordinations with Bray–Curtis dissimilarity measures. The significance of the various sources of variation in the data was calculated using ANOSIM and NPMANOVA with *P* values less than 0.05 considered significant. The significance was computed by permutation of group membership, with 9999 replicates. NPMANOVA was used for the pairwise comparisons of the bacterial communities in the MR and MS colonies across the season, as well as for each sampling occasion. The differences in the RT-qPCR derived viral titres between the MR and MS populations for each sampling occasion were analysed using Welch's t-test, with *P* values less than 0.05 considered significant. All statistical analyses were performed using the PAST software^90^.

## Supplementary Information

Supplementary Information.

## Data Availability

The datasets generated during and/or analysed during the current study are available from the corresponding author on reasonable request.
