# Supplementary material for: Global similarity, and some key differences, in the metagenomes of Swedish varroa-surviving and varroa-susceptible honeybees
Source: Sci Rep. 2021 Dec 1;11:23214. doi: 10.1038/s41598-021-02652-x (PMC8636477; doi:10.1038/s41598-021-02652-x)
Supplement: Supplementary file 1 — Supplementary Information. [file 41598_2021_2652_MOESM1_ESM.docx]

**Supplementary Files**

Global equivalency, and some key differences, in the metagenomes of Swedish varroa-surviving and varroa-susceptible honeybees

Srinivas Thaduri, Srisailam Marupakula, Olle Terenius, Piero Onorati, Christian Tellgren-Roth, Barbara Locke, Joachim R. de Miranda

1. PHYLUM


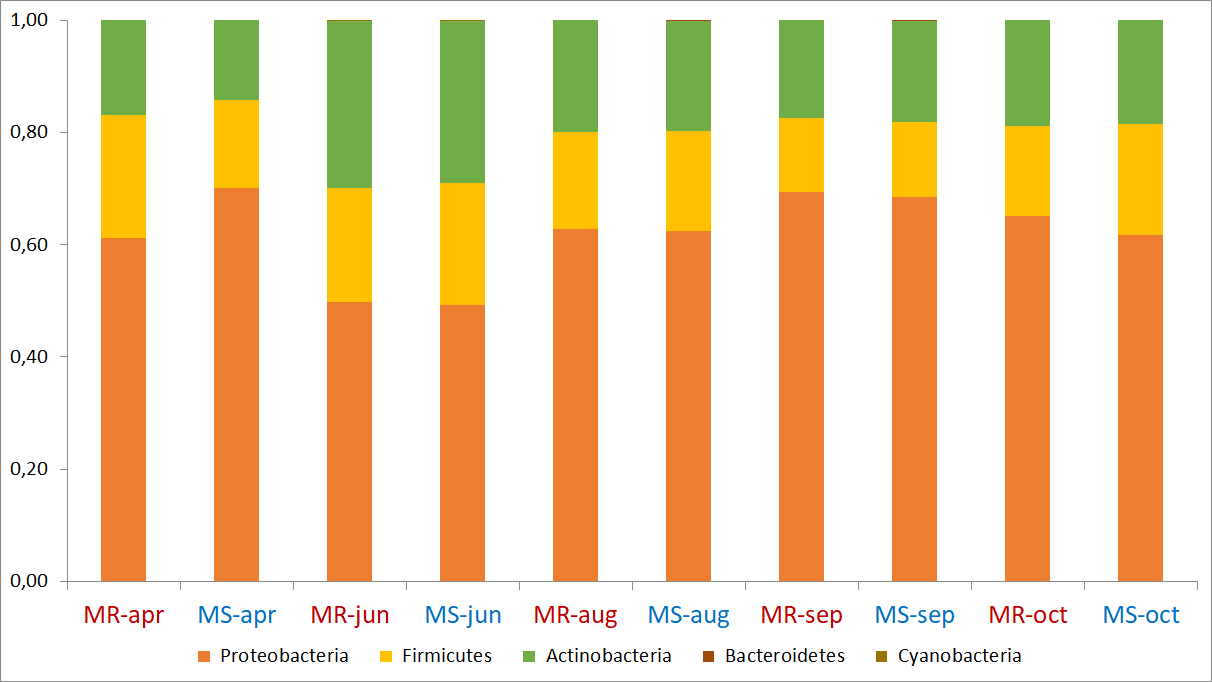


1. CLASS


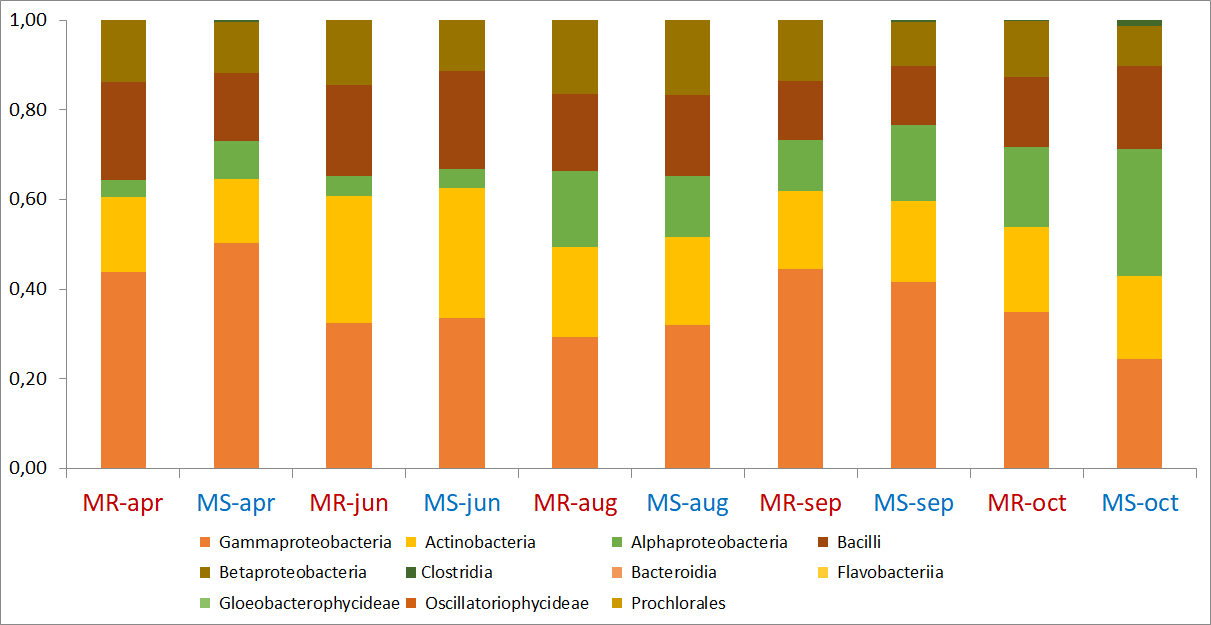


**Supplementary Figure 1: Bacterial community composition.**

Taxonomic distribution of the sequence reads for the V2 hypervariable region of the bacterial 16S rDNA gene obtained for the bi-monthly samples of the MR and MS colonies, as determined at Phylum (A) and Class (B) level.


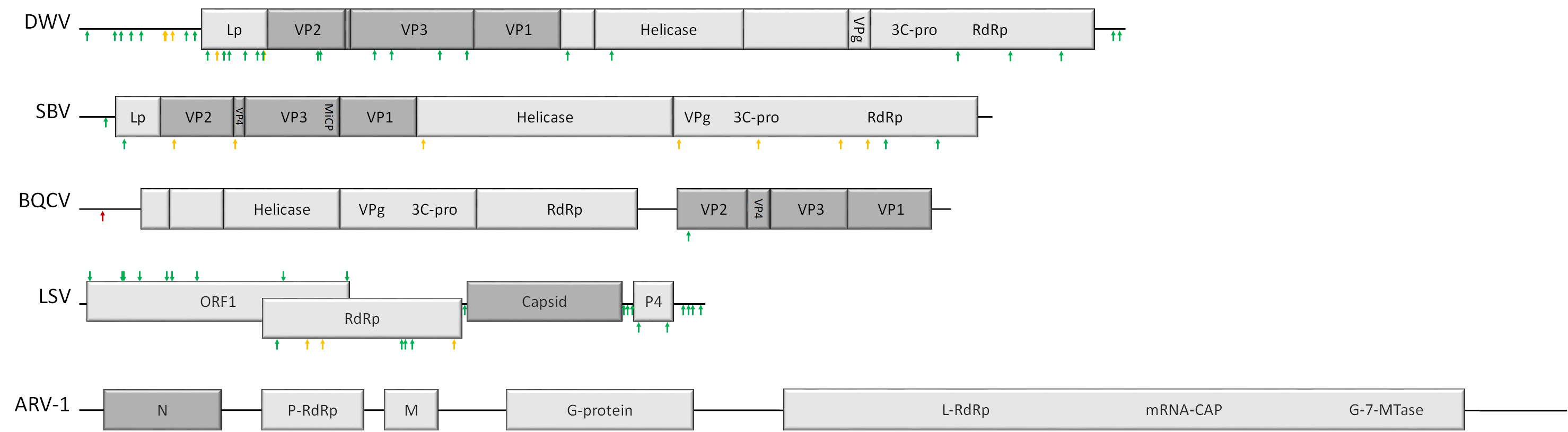


**Supplementary Figure 2: Distribution of seasonal and population-related variants across viral genomes.**

Genome maps of the five viruses in the study (DWV, SBV, BQCV, LSV, ARV-1) showing the different structural (dark grey) and non-structural (light grey) proteins. The DWV, SBV and BQCV structural proteins are numbered according to ICTV convention^89,90^. For DWV, SBV and BQCV, whose genome expression involves proteolytic cleavage of polyproteins, the different protein subunits were identified by the putative 3C-proteolytic processing sites^91^. The coloured arrows show the locations of those genetic variations in the nucleotide (non-coding regions) and amino acid (coding regions) sequences that are consistently linked to either seasonality, equally in both populations (green), population of origin, consistently throughout the year (red) or a combination of both, i.e. seasonality in only one population (yellow). The following functional proteins/domains are identified: L-protein (Lp), viral proteins 1, 2, 3 and 4 (VP1, VP2, VP3, VP4), including the MiCP peptide of VP3^91^; the helicase; the viral protein genome-linked (VPg); The 3C protease (3C-pro); the RNA-dependent RNA polymerase (RdRp); the capsid protein; unknown protein P4; the nucleoprotein (N); the P- and L-subunits of the RNA dependent RNA polymerase (P-RdRp and L-RdRp); a putative movement protein (M); a glycol-protein (G-protein); an mRNA capping protein (mRNA-CAP) and a G-7 methyl transferase (G-7-MTase). Structural proteins are coloured in dark grey, non-structural proteins in light grey.


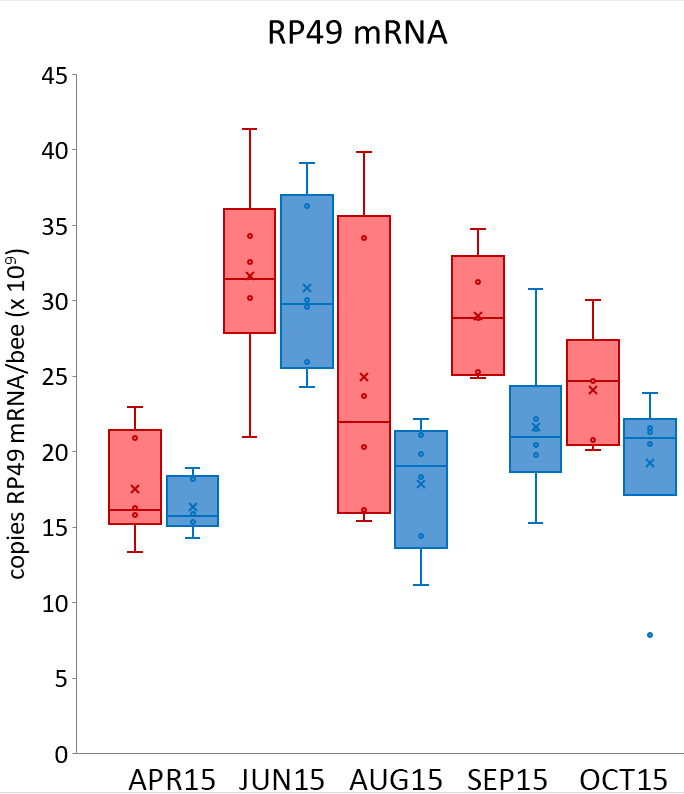


**Supplementary Figure 3: RP49 levels.**

Box plots of the amounts of RP49 mRNA detected in the adult bee samples from the MR (red) and MS (blue) colonies during the 2015 season, as determined by RT-qPCR.

**
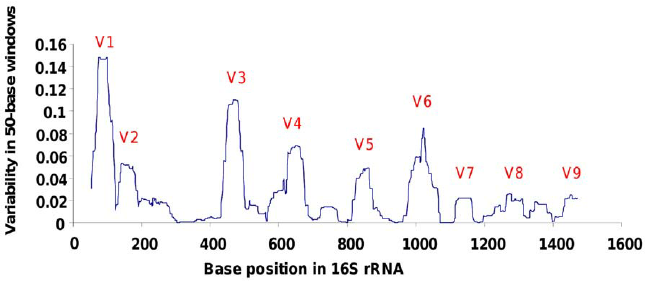
**

**Supplementary Figure 4: Variable regions bacterial 16S rDNA gene.**

Map of the relative genome positions of hypervariable regions of the bacterial 16S rDNA gene, based on the map for *Pseudomonas*. After Bodilis *et al.*^92^.

**
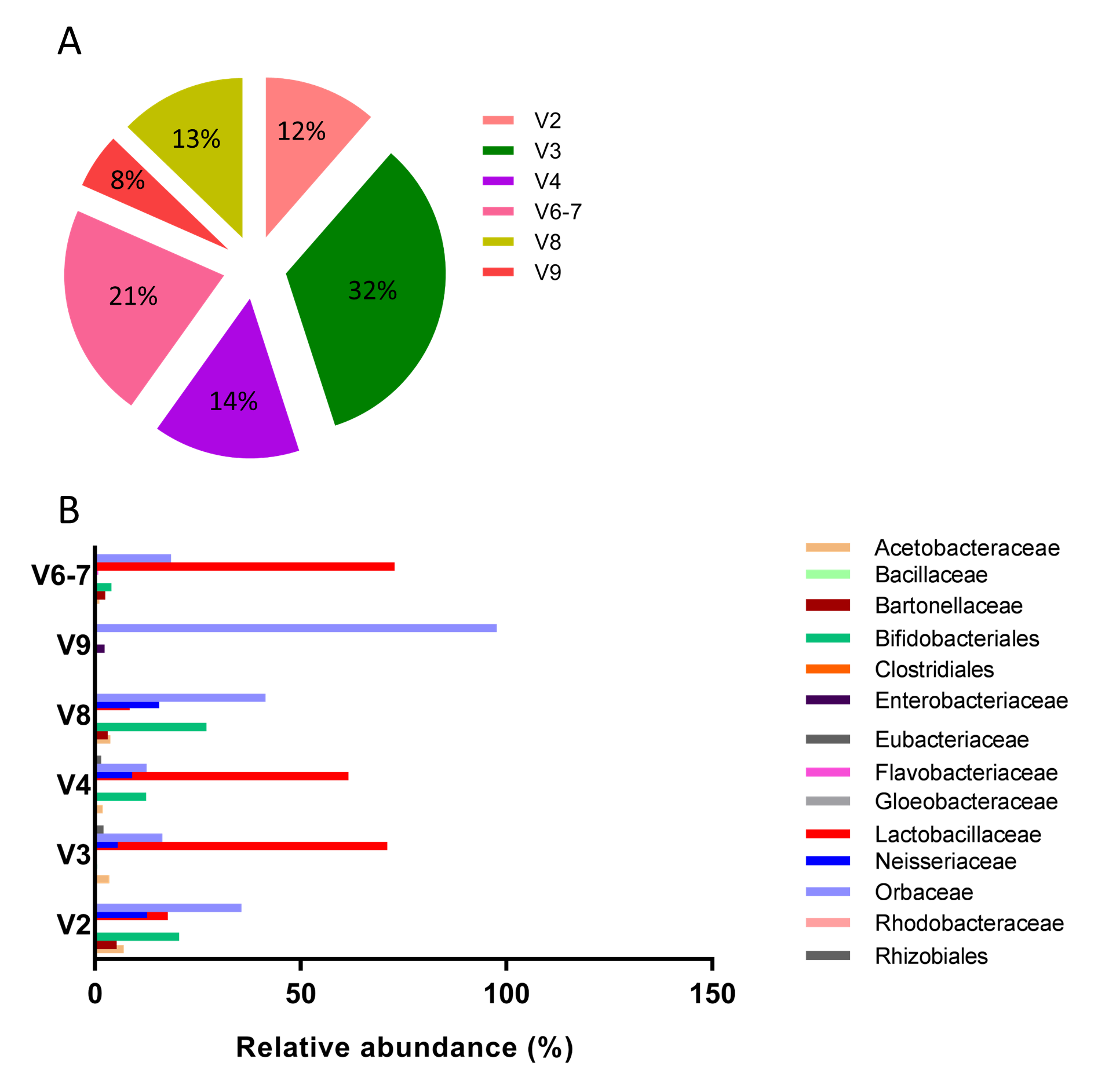
**

**Supplementary Figure 5: Efficiency analysis 16S rDNA variable regions.**

Efficiency of seven hypervariable regions (V2 –V9) of the 16S-rRNA gene for genus and species identification of bacteria. (A) Division of total read counts obtained for the different hypervariable regions. (B) Distribution of mapped reads among the different hypervariable regions among different bacterial families, shown as the percentage of read counts mapping to the different bacterial taxa, calculated separately for each hypervariable region.

**
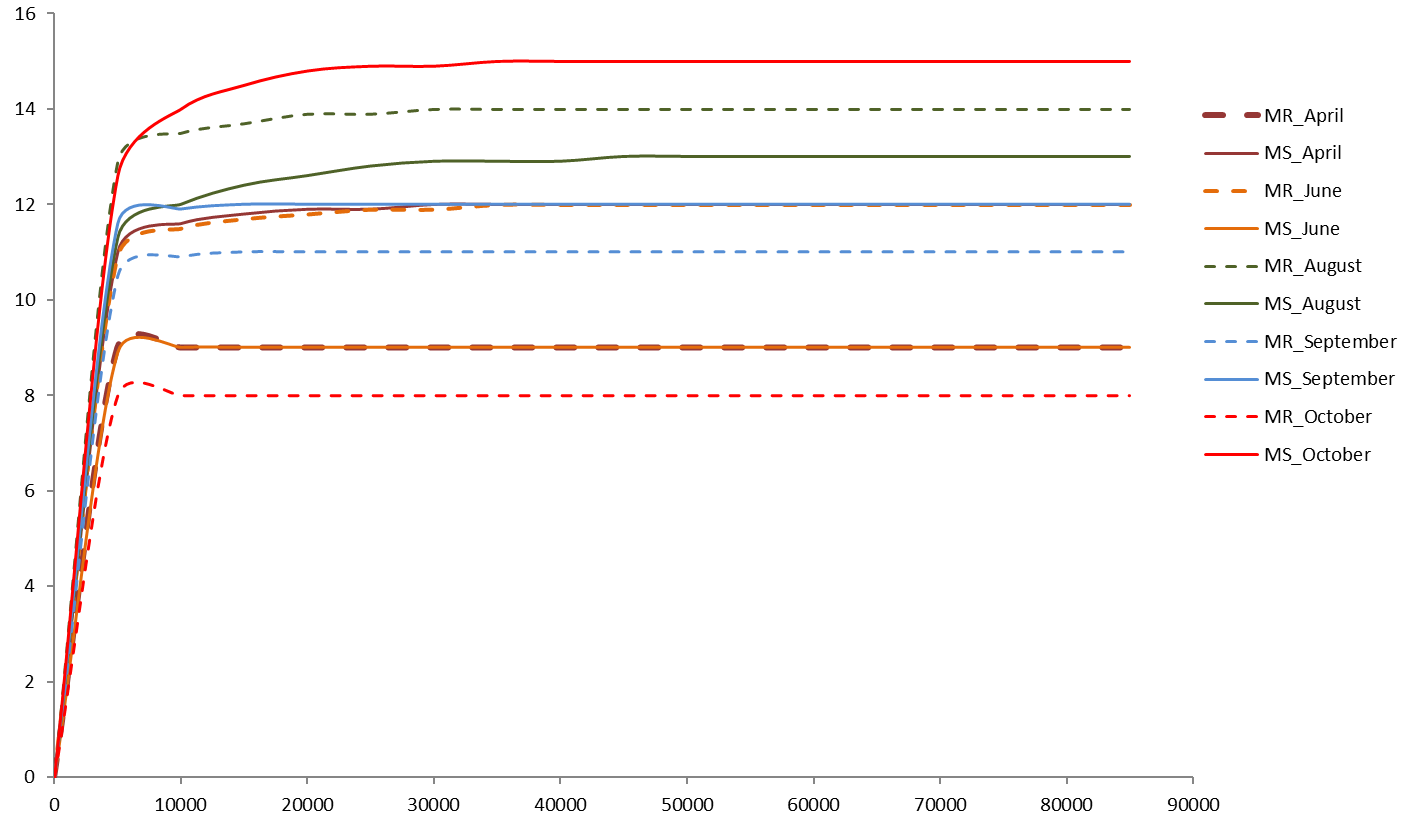
**

**Supplementary Figure 6: Rarefaction saturation curves bacterial analyses.**

Rarefaction saturation curves for the taxonomic diversity of the bacterial communities of the MR and MS populations at each of the five sampling occasions, based on the V2 hypervariable region of the bacterial 16S rDNA.

| **Index** | **APR15** | | | **JUN15** | | | **AUG15** | | | **SEP15** | | | **OCT15** | | |
| --- | --- | --- | --- | --- | --- | --- | --- | --- | --- | --- | --- | --- | --- | --- | --- |
|  | **MR** | **MS** | ***P*^0.05^** | **MR** | **MS** | ***P*^0.05^** | **MR** | **MS** | ***P*^0.05^** | **MR** | **MS** | ***P*^0.05^** | **MR** | **MS** | ***P*^0.05^** |
| **Richness** |  |  |  |  |  |  |  |  |  |  |  |  |  |  |  |
| Taxa S | 9 | 12 |  | 12 | 9 |  | 14 | 13 |  | 11 | 12 |  | 8 | 15 |  |
| Menhinick | 0,03 | 0,04 |  | 0,04 | 0,03 |  | 0,05 | 0,04 |  | 0,04 | 0,04 |  | 0,03 | 0,05 |  |
| Margalef | 0,70 | 0,97 |  | 0,97 | 0,70 |  | 1,14 | 1,06 |  | 0,88 | 0,97 |  | 0,62 | 1,23 |  |
| **Evenness** |  |  |  |  |  |  |  |  |  |  |  |  |  |  |  |
| Dominance D | 0,23 | 0,25 | * | 0,22 | 0,22 |  | 0,17 | 0,17 |  | 0,19 | 0,18 | * | 0,19 | 0,17 | * |
| Berger-Parker | 0,37 | 0,44 | * | 0,30 | 0,29 |  | 0,24 | 0,24 |  | 0,33 | 0,32 | * | 0,30 | 0,20 | * |
| Evenness_e^H/S | 0,57 | 0,44 | * | 0,43 | 0,58 | * | 0,47 | 0,50 | * | 0,55 | 0,53 | * | 0,71 | 0,44 | * |
| Equitability_J | 0,74 | 0,67 | * | 0,66 | 0,75 | * | 0,71 | 0,73 | * | 0,75 | 0,74 | * | 0,84 | 0,70 | * |
| **Diversity Index** |  |  |  |  |  |  |  |  |  |  |  |  |  |  |  |
| Shannon H | 1,63 | 1,66 | * | 1,64 | 1,65 | * | 1,88 | 1,88 |  | 1,80 | 1,85 | * | 1,74 | 1,88 | * |
| Simpson 1-D | 0,77 | 0,75 | * | 0,78 | 0,78 |  | 0,83 | 0,83 |  | 0,81 | 0,82 | * | 0,81 | 0,83 | * |
| Fisher's alpha | 0,77 | 1,06 |  | 1,06 | 0,77 |  | 1,26 | 1,16 |  | 0,96 | 1,06 |  | 0,68 | 1,36 |  |

**Supplementary Table 1: Biodiversity analyses bacterial composition.**

Selection of biodiversity measures for the bacterial communities in the seasonal samples of the MR and MS colonies, separated into three categories: species ‘richness’, the ‘evenness’ of the frequency distributions of these species, and common diversity ‘indices’ that combine different weights of ‘richness’ and ‘evenness’. The significance at P < 0.05 of the difference between the MR and MS colonies at each sampling occasion for each of the measures is indicated by an asterisk (*).

|  |  | **MS** | | | | | **MR** | | | | |
| --- | --- | --- | --- | --- | --- | --- | --- | --- | --- | --- | --- |
|  |  | **APR15** | **JUN15** | **AUG15** | **SEP15** | **OCT15** | **APR15** | **JUN15** | **AUG15** | **SEP15** | **OCT15** |
| **MS** | **APR15** | * | 0.011 | 0.008 | 0.059 | 0.006 | 0.067 | 0.004 | 0.013 | 0.050 | 0.017 |
|  | **JUN15** | 0.011 | * | 0.074 | 0.002 | 0.003 | 0.036 | 0.922 | 0.015 | 0.013 | 0.004 |
|  | **AUG15** | 0.008 | 0.074 | * | 0.095 | 0.009 | 0.013 | 0.047 | 0.948 | 0.050 | 0.008 |
|  | **SEP15** | 0.059 | 0.002 | 0.095 | * | 0.003 | 0.002 | 0.003 | 0.053 | 0.580 | 0.028 |
|  | **OCT15** | 0.006 | 0.003 | 0.009 | 0.003 | * | 0.002 | 0.003 | 0.003 | 0.016 | 0.214 |
| **MR** | **APR15** | 0.067 | 0.036 | 0.013 | 0.002 | 0.002 | * | 0.002 | 0.009 | 0.006 | 0.002 |
|  | **JUN15** | 0.004 | 0.922 | 0.047 | 0.003 | 0.003 | 0.002 | * | 0.026 | 0.004 | 0.002 |
|  | **AUG15** | 0.013 | 0.015 | 0.948 | 0.053 | 0.003 | 0.009 | 0.026 | * | 0.031 | 0.020 |
|  | **SEP15** | 0.050 | 0.013 | 0.050 | 0.580 | 0.016 | 0.006 | 0.004 | 0.031 | * | 0.047 |
|  | **OCT15** | 0.017 | 0.004 | 0.008 | 0.028 | 0.214 | 0.002 | 0.002 | 0.020 | 0.047 | * |

**Supplementary Table 2: Multivariate analyses bacterial community structure.**

Non-parametric multivariate analysis of variance (NPMANOVA) P values for pairwise comparisons between MR and MS colonies at different times during the season. Values are the P-values representing the significance of the differences in bacterial community structure in the pairwise comparison. High P-values represent highly similar microbial communities.

|  | **SBV** | | | | |
| --- | --- | --- | --- | --- | --- |
|  | **mean^MR^** | **mean^MS^** | ***t_(MR-MS)_*** | **df** | ***P*** |
| **APR15** | 6.65 | 8.88 | -3.03 | 9.47 | **0.013** |
| **JUN15** | 9.49 | 9.91 | -0.70 | 6.70 | 0.505 |
| **AUG15** | 9.55 | 9.42 | 0.144 | 9.99 | 0.888 |
| **SEP15** | 6.38 | 8.13 | -2.07 | 7.57 | 0.073 |
| **OCT15** | 5.94 | 7.61 | -2.22 | 5.74 | 0.069 |

**Supplementary Table 3: SBV quantitative analyses.**

Results of Welch’s t-test testing the differences between the MR and MS colonies in SBV virus titres for the five sampling occasions in 2015.

| **RT-qPCR assay** | **Primers** | **Sequence (5' - 3')** | **Size** | **r^2^** | ***T_m_*** |
| --- | --- | --- | --- | --- | --- |
| DWV | DWV-F8668 | TTCATTAAAGCCACCTGGAACATC | 136 | 0.992 | 79.5 ^o^C |
|  | DWV-B8757 | TTTCCTCATTAACTGTGTCGTTGA |  |  |  |
| SBV | SBV-qF3164 | TTGGAACTACGCATTCTCTG | 335 | 0.974 | 80.3 ^o^C |
|  | SBV-qB3461 | GCTCTAACCTCGCATCAAC |  |  |  |
| BQCV | BQCV-qF7893 | AGTGGCGGAGATGTATGC | 294 | 0.990 | 80.5 ^o^C |
|  | BQCV-qB8150 | GGAGGTGAAGTGGCTATATC |  |  |  |
| LSV | LSV-qF3025 | GTCTCGTTGCAGTGGTGACC | 160 | 0.998 | 82.0 ^o^C |
|  | LSV-qB3185 | GTAGTGCTCCACCTTAAGC |  |  |  |
| ARV-1 | ARV-qF9110 | TCTGGCAATTCATGATCCTCCA | 152 | 0.981 | 79.0 ^o^C |
|  | ARV-qB9262 | AAGATGTCCAGGTCTCTGCG |  |  |  |
| RP49 mRNA | RP49-qF | AAGTTCATTCGTCACCAGAG | 205 | 0.999 | 77.6 ^o^C |
|  | RP49-qB | CTTCCAGTTCCTTGACATTATG |  |  |  |

**Supplementary Table 4: Summary RT-qPCR assays.**

Details of the diagnostic RT-qPCR assays used for the five viruses (DWV, SBV, BQCV, LSV, ARV-1) and the internal reference gene (RP49), including primer names and sequences, amplicon size, linearity of the external calibration curves over 6 orders of magnitude (r^2^) and the mean temperature of the main peak of the Melting Curve (*T_m_*). All assays are broadly consensual for the main variants and strains of each virus.

| **Sample** | | **Raw** | **Mapped Nucleotides** | | | | |
| --- | --- | --- | --- | --- | --- | --- | --- |
|  |  | **reads** | **DWV** | **SBV** | **BQCV** | **LSV** | **ARV-1** |
| **MR** | APR15 | 30 015 504 | 37 466 | 181 432 | 5 614 553 | 11 547 080 | 151 993 |
|  | JUN15 | 27 163 622 | 103 517 | 30 044 797 | 10 797 274 | 56 791 349 | 78 425 |
|  | AUG15 | 30 174 009 | 738 142 | 23 867 940 | 2 333 110 | 9 730 718 | 73 256 |
|  | SEP15 | 30 366 744 | 395 458 170 | 25 733 | 4 911 716 | 23 866 | 813 617 |
|  | OCT15 | 31 039 679 | 685 018 857 | 17 876 | 33 385 156 | 17 323 | 84 201 |
| **MS** | APR15 | 28 535 216 | 1 761 540 | 19 142 298 | 3 007 678 | 10 201 231 | 33 417 |
|  | JUN15 | 30 093 880 | 94 189 | 55 480 981 | 13 186 589 | 9 717 472 | 105 604 |
|  | AUG15 | 31 122 695 | 37 752 390 | 25 409 048 | 3 143 011 | 4 676 833 | 138 414 |
|  | SEP15 | 28 618 484 | 114 578 551 | 8 373 514 | 7 894 852 | 36 440 | 104 511 |
|  | OCT15 | 32 474 292 | 238 756 456 | 9 722 190 | 29 072 678 | 383 366 | 72 812 |
| Accession | | | MH267695 | MH267697 | MH267693 | MH267699 | MH267691 |
| Genome size (nt) | | | 10152 | 8830 | 8432 | 5977 | 14583 |

**Supplementary Table 5: RNA sequencing and mapping data.**

Numerical details of the bioinformatic analyses of the RNA sequencing data. Shown are the total number of raw reads obtained for each sample, the number of nucleotides from these reads that mapped to the DWV, SBV, BQCV, LSV and ARV-1 reference genomes and the GenBank accession numbers and sizes of these reference genomes.

| **Phylogenetic analyses** | | | | **Accession Numbers** | | | | | |
| --- | --- | --- | --- | --- | --- | --- | --- | --- | --- |
| **Virus** | **ML estimate (log)** | **Characters** | **Taxa** | **MR colonies** | **Accession** | **MS colonies** | **Accession** | **Reference Genomes** | **Accession** |
| Deformed wing virus | -30012.24 | 10072 | 15 | ***MR-apr15*** | *MT636317* | ***MS-apr15*** | *MT636322* | DWV-MR | MH267695 |
|  |  |  |  | ***MR-jun15*** | *MT636318* | ***MS-jun15*** | *MT636323* | DWV-MS | MH267696 |
|  |  |  |  | ***MR-aug15*** | *MT636319* | ***MS-aug15*** | *MT636324* | DWV-A | AY292384 |
|  |  |  |  | ***MR-sep15*** | *MT636320* | ***MS-sep15*** | *MT636325* | DWV-B | AY251269 |
|  |  |  |  | ***MR-oct15*** | *MT636321* | ***MS-oct15*** | *MT636326* | DWV-C | CEND01000001 |
|  |  |  |  |  |  |  |  | DWV-D | MT504363 |
| Sacbrood virus | -25416.27 | 8782 | 16 | ***MR-apr15*** | *MT636327* | ***MS-apr15*** | *MT636332* | SBV-MR | MH267697 |
|  |  |  |  | ***MR-jun15*** | *MT636328* | ***MS-jun15*** | *MT636333* | SBV-MS | MH267698 |
|  |  |  |  | ***MR-aug15*** | *MT636329* | ***MS-aug15*** | *MT636334* | SBV-uk | AF092924 |
|  |  |  |  | ***MR-sep15*** | *MT636330* | ***MS-sep15*** | *MT636335* | SBV-cn | HM237361 |
|  |  |  |  | ***MR-oct15*** | *MT636331* | ***MS-oct15*** | *MT636336* | SBV-th | KM884995 |
|  |  |  |  |  |  |  |  | SBV-ko | JQ390591 |
| Black queen cell virus | -21298.47 | 8378 | 15 | ***MR-apr15*** | *MT636337* | ***MS-apr15*** | *MT636342* | BQCV-MR | MH267693 |
|  |  |  |  | ***MR-jun15*** | *MT636338* | ***MS-jun15*** | *MT636343* | BQCV-MS | MH267694 |
|  |  |  |  | ***MR-aug15*** | *MT636339* | ***MS-aug15*** | *MT636344* | BQCV-sa | AF183905 |
|  |  |  |  | ***MR-sep15*** | *MT636340* | ***MS-sep15*** | *MT636345* | BQCV-cz | KY243932 |
|  |  |  |  | ***MR-oct15*** | *MT636341* | ***MS-oct15*** | *MT636346* | BQCV-cn | KY741959 |
| Lake Sinai virus | -23767.38 | 5350 | 15 | ***MR-apr15*** | *MT636347* | ***MS-apr15*** | *MT636352* | LSV-MR | MH267699 |
|  |  |  |  | ***MR-jun15*** | *MT636348* | ***MS-jun15*** | *MT636353* | LSV-MS | MH267700 |
|  |  |  |  | ***MR-aug15*** | *MT636349* | ***MS-aug15*** | *MT636354* | LSV-1 | KY465697 |
|  |  |  |  | ***MR-sep15*** | *MT636350* | ***MS-sep15*** | *MT636355* | LSV-2 | KY465706 |
|  |  |  |  | ***MR-oct15*** | *MT636351* | ***MS-oct15*** | *MT636356* | LSV-3 | KY465717 |
| Apis rhabdovirus-1 | -22125.42 | 13824 | 17 |  |  |  |  | ARV1-MR | MH267691 |
|  |  |  |  | ***MR-apr15*** | *MT636357* | ***MS-apr15*** | *MT636362* | ARV1-MS | MH267692 |
|  |  |  |  | ***MR-jun15*** | *MT636358* | ***MS-jun15*** | *MT636363* | ARV-nl | KY354230 |
|  |  |  |  | ***MR-aug15*** | *MT636359* | ***MS-aug15*** | *MT636364* | ARV-sa | KY354231 |
|  |  |  |  | ***MR-sep15*** | *MT636360* | ***MS-sep15*** | *MT636365* | ARV-to | KY354232 |
|  |  |  |  | ***MR-oct15*** | *MT636361* | ***MS-oct15*** | *MT636366* | ARV-us | MF114349 |
|  |  |  |  |  |  |  |  | ARV-il | MF114351 |

**Supplementary Table 6: Numerical summary of the phylogenetic analyses.**

Supplementary data and information underlying the phylogenetic analyses, including the ML estimate of the optimum tree, the number of characters and taxa included in the reconstruction, as well as the GenBank accession numbers of the full-length consensus sequences of the experimental and outgroup sequences used in the bioinformatic and phylogenetic analyses.

**Supplementary references**

1. Valles, S.M. et al. ICTV virus taxonomy profile: Iflaviridae. *J. Gen. Virol.* **98**, 527-528 (2017).
2. Valles, S.M. et al. ICTV virus taxonomy profile: Dicistroviridae. *J. Gen. Virol.* **98**, 355-356 (2017).
3. Procházková, M. et al. Virion structures and genome delivery of honeybee viruses. *Curr. Opin. Virol.* **45**, 17–24 (2020).
4. Bodilis, J., Nsigue-Meilo, S., Besaury, L. & Quillet. L. Variable copy number, intra-genomic heterogeneities and lateral transfers of the 16S rRNA gene in pseudomonas. *PLoS ONE* **7**, e35647 (2012).
